# Nonconvulsive Status Epilepticus With Generalized Spike-and-Wave Discharges: Pathophysiological and Nosological Considerations

**DOI:** 10.7759/cureus.47401

**Published:** 2023-10-20

**Authors:** Madison C Wolf, Kristen S Butner, Elizabeth B Brinkley, Joshua B Campo, Piotr Olejniczak, Edward C Mader

**Affiliations:** 1 Electrodiagnostic Technology, LCMC Health, New Orleans, USA; 2 Neurology, LCMC Health, New Orleans, USA; 3 Neurology, Louisiana State University (LSU) Health Sciences Center, New Orleans, USA

**Keywords:** thalamocortical, frontal lobe epilepsy, de novo, dialeptic, absence, nonconvulsive, status epilepticus, spike-and-wave

## Abstract

Absence status epilepticus (ASE) is the most common type of status epilepticus in patients with idiopathic generalized epilepsy (IGE). Like absence seizure, ASE is characterized by generalized spike-and-wave discharges (GSWDs) on the electroencephalogram (EEG). Once considered specific for IGE, GSWDs have increasingly been observed in other forms of epilepsy, as well as in patients with no prior epilepsy. Here, we report three patients with different types of nonconvulsive status epilepticus (NCSE) in which the EEG correlate was GSWDs: a 44-year-old woman with juvenile absence epilepsy who manifested ASE, a 73-year-old woman with anoxic brain injury complicated by NCSE with well-formed GSWDs (as seen in IGE and de novo ASE), and a 41-year-old woman with frontal lobe epilepsy who developed focal NCSE with impaired consciousness. Evidently, patients with generalized epilepsy, focal epilepsy, and no prior epilepsy can all manifest NCSE with similar electroclinical characteristics, i.e., GSWDs and impaired consciousness. This observation adds to the existing evidence that seizures, whether classified as focal or generalized, often involve focal and generalized elements in their pathophysiology. To fully understand seizure pathophysiology, we must steer away from the focal-versus-generalized paradigm that has dominated the nosology of seizures and epilepsy for a very long time.

## Introduction

Generalized spike-and-wave discharges (GSWDs) are seen in the electroencephalogram (EEG) of patients with idiopathic (genetic) generalized epilepsy (IGE) [1]. GSWDs may appear as generalized polyspike-and-wave discharges. This pattern and other variations of GSWDs are simply referred to as GSWDs in this article. Ictal and interictal GSWDs are highly characteristic of childhood absence epilepsy and juvenile absence epilepsy [2]. GSWDs may also be seen on the scalp EEG of patients with other IGE syndromes, such as juvenile myoclonic epilepsy, Jeavons syndrome, and Doose syndrome [3]. In contrast to IGE, focal epilepsy is not usually expressed as GSWDs and, whenever GSWDs are present in the EEG of patients with focal epilepsy, the discharges are often poorly formed and tend to lateralize towards the hemisphere with the seizure focus [4]. Hence, the detection of GSWDs in a patient with a clinical diagnosis of focal epilepsy often encourages the physician to search for an alternative epilepsy diagnosis [5].

The International League Against Epilepsy (ILAE) proposed a quadriaxial system for classifying status epilepticus [6]. Status epilepticus is categorized based on the following information (referred to as “axes”): semiology, etiology, EEG correlate, and patient age. Axis 1 (semiology) divides nonconvulsive status epilepticus (NCSE) into NCSE with coma and NCSE without coma. NCSE without coma can either be generalized, focal, or unknown whether focal or generalized. Generalized NCSE includes typical, atypical, and myoclonic absence status epilepticus (ASE). Focal NCSE includes NCSE with and NCSE without impaired consciousness [6]. Focal NCSE with impaired consciousness, formerly known as complex partial status epilepticus (CPSE), is the most common type of NCSE in patients with focal epilepsy [7]. However, in patients with IGE, ASE is the most common form of NCSE [8]. ASE is characterized by GSWDs and loss of awareness with subtle motor features [9]. ASE may also appear “de novo” in patients with or without a history of absence epilepsy and, like typical ASE, de novo ASE is expressed as GSWDs in the EEG [10].

Here, we present a report on three patients whose EEG showed GSWDs during an episode of NCSE: a 44-year-old woman with juvenile absence epilepsy who manifested ASE, a 73-year-old woman with anoxic brain injury complicated by de novo ASE, and a 41-year-old woman with frontal lobe epilepsy who developed focal NCSE with impaired consciousness.

## Case presentation

**Figure 1:**
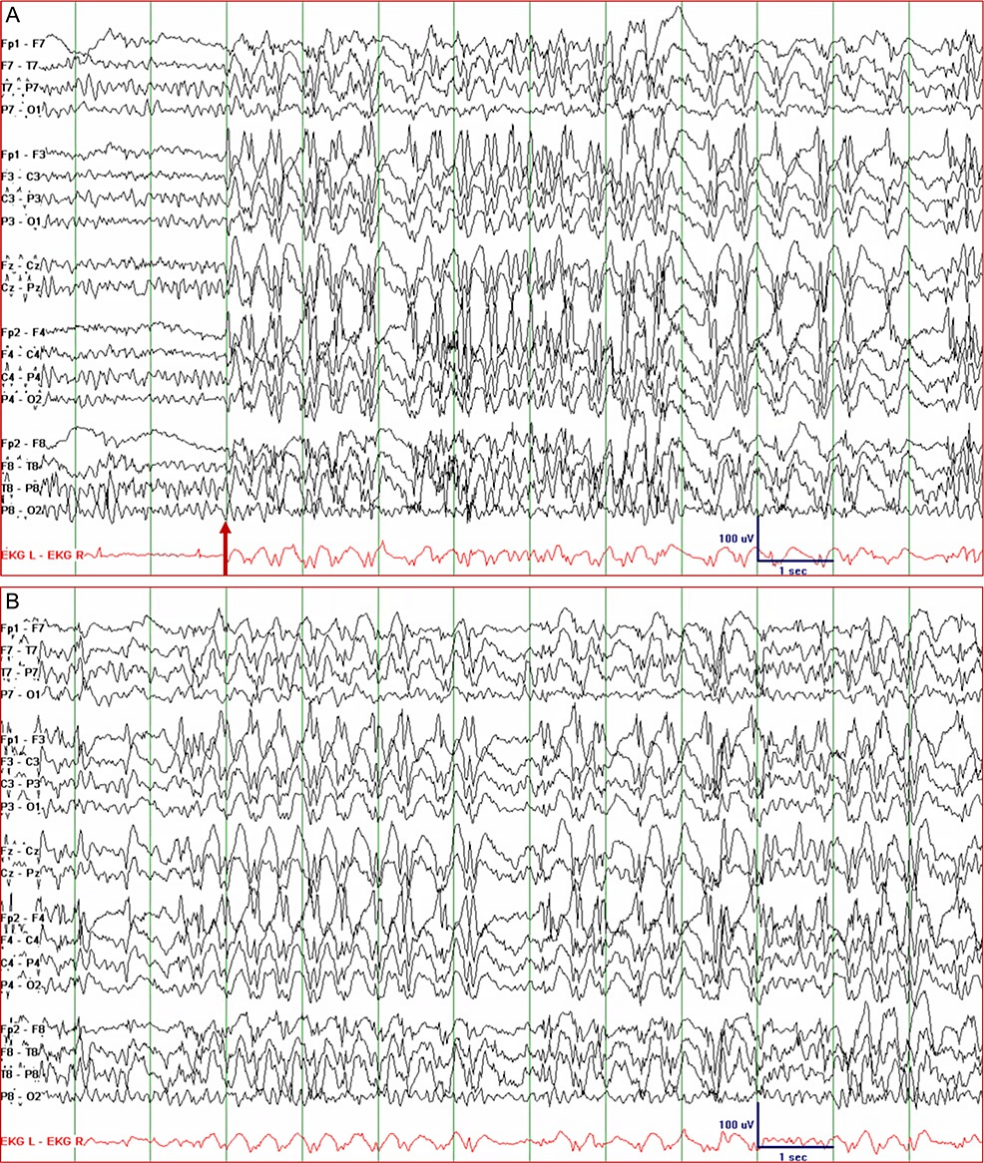
Electroencephalogram of Patient 1 showing absence status epilepticus. (A) Generalized spike-and-wave discharges with a frequency of 2.5-3 Hz appeared abruptly (arrow). (B) The discharges persisted for about 52 minutes with minimal fluctuations in amplitude and frequency during which the patient had impairment of concentration, intermittent unresponsiveness, and constant blinking.

Patient 1 was a 44-year-old woman with intractable juvenile absence epilepsy. At the age of 13, she started having absences and myoclonic seizures and, at the age of 19, she also started having generalized tonic-clonic seizures. She tried almost all antiseizure drugs in various combinations and at maximal dosages and had been on vagus nerve stimulation (VNS) therapy since the age of 22. Despite these measures, she continued to have absence seizures every day, myoclonic seizures every few weeks, and generalized tonic-clonic seizures two to three times a year. Deep brain stimulation was being considered, so she was admitted to the epilepsy monitoring unit (EMU). While off most of her antiseizure drugs, her EEG showed 2.5-3 Hz GSWDs that gradually became more frequent and longer in duration. On day 2, she lapsed into a state of inattention and intermittent unresponsiveness. She was also constantly blinking. Her EEG showed continuous 2.5-3.5 Hz GSWDs with waxing and waning amplitude for about 52 minutes (Figure 1). ASE stopped immediately after the administration of lorazepam 1 mg IV. Her antiseizure drugs were resumed and she did not have any more seizures in the EMU.

**Figure 2:**
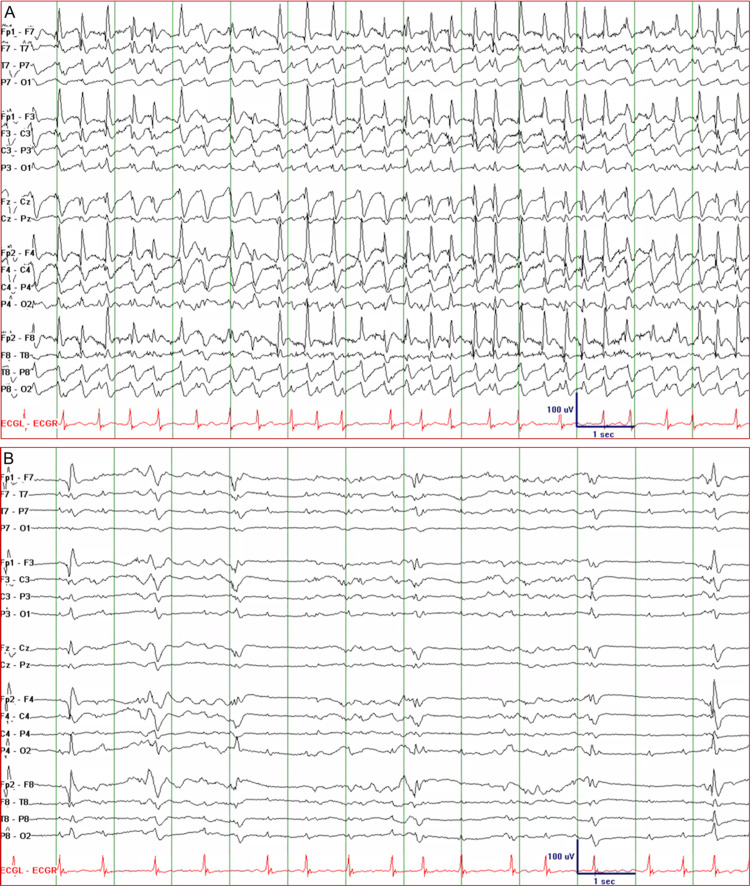
Electroencephalogram of Patient 2 showing nonconvulsive status epilepticus. (A) The tracing shows well-formed generalized spike-and-wave discharges with a frequency of 2.5-3 Hz suggestive of de novo absence status epilepticus. (B) The epileptiform discharges started to break up after the administration of 2 mg lorazepam IV but re-emerged later; they were completely suppressed with a propofol IV infusion rate of 8 mg/kg/h.

Patient 2 was a 73-year-old woman with a history of stroke, congestive heart failure, and deep vein thrombosis, but no prior history of seizure or epilepsy. She was found down at home in ventricular fibrillation. She was successfully resuscitated, intubated, and admitted to the intensive care unit with a Glasgow Coma Scale (GCS) score of 6. EEG was immediately recorded, and this initially showed well-formed 2.5-3 Hz GSWDs consistent with de novo ASE (Figure 2, top tracing). GSWDs started to break up with 2 mg lorazepam IV (Figure 2, bottom tracing). Levetiracetam 2000 mg IV load, then 1000 mg q12 and propofol IV infusion at a rate of 2 mg/kg/h were started. Continuous EEG showed reemerging GSWDs, so propofol was uptitrated to 5 mg/kg/h IV infusion and lacosamide 100 mg IV q12h was added. GSWDs started to break up and were completely suppressed when the propofol infusion rate was increased to 8 mg/kg/h. There was no recurrence of GSWDs, so propofol was tapered and discontinued. The patient was maintained on levetiracetam and lacosamide. Her GCS score increased to 8 and she was transferred to another facility for palliative care.

**Figure 3:**
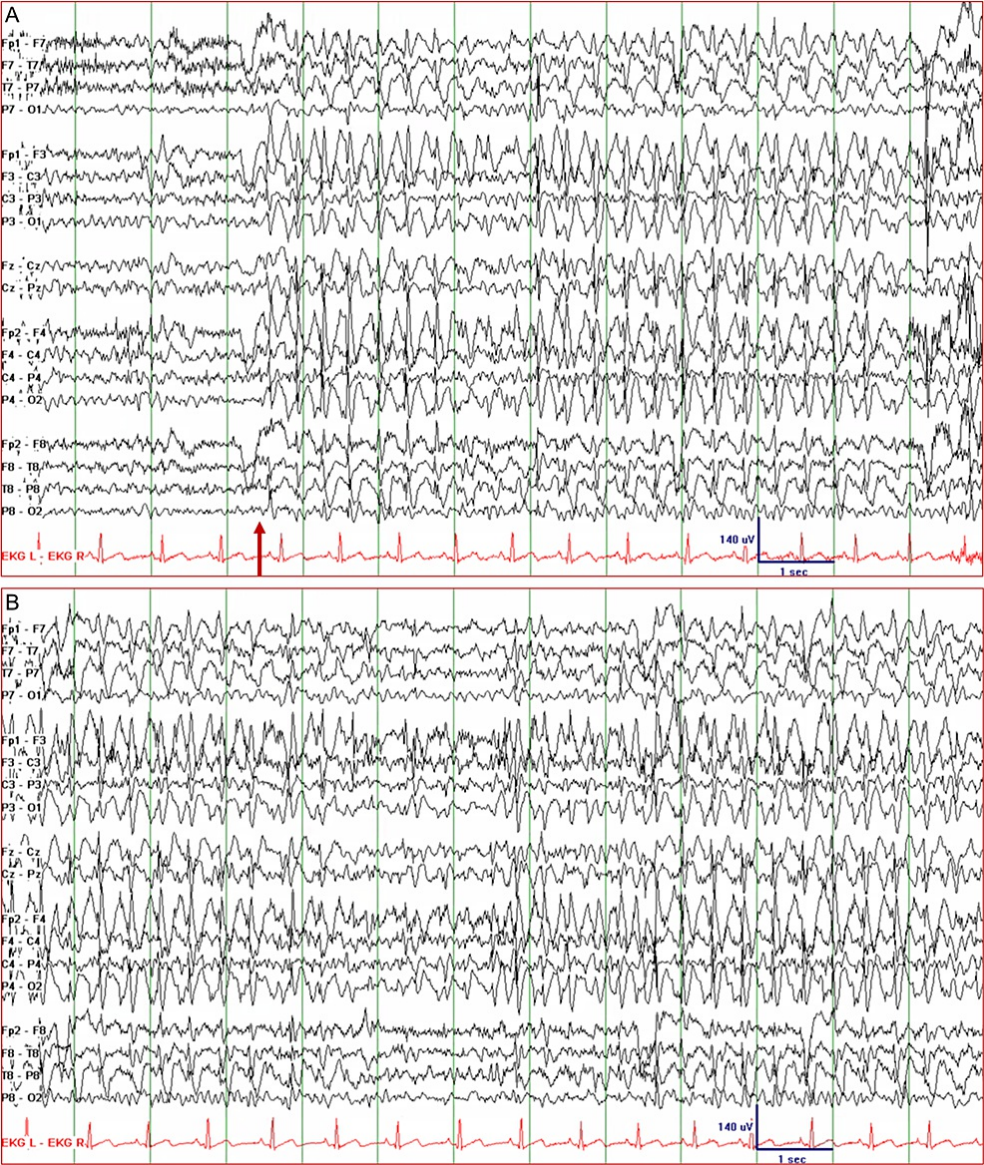
Electroencephalogram of Patient 3 showing nonconvulsive status epilepticus. (A) Generalized spike-and-wave discharges with a frequency of 2.5-3.5 Hz appeared simultaneously over both hemispheres (arrow), although the seizure may have originated from a focus in one frontal lobe. (B) The seizure persisted for about 36 minutes during which the patient was confused and disoriented with verbal output limited to one- or two-word responses.

Patient 3 was a 41-year-old woman with intractable frontal lobe epilepsy and normal brain magnetic resonance imaging studies. She started having focal seizures and generalized tonic-clonic seizures at the age of 18. Her focal seizures were characterized by leftward head-turning, fencing posture (left arm extended, right arm flexed), and occasional vocalizations, with or without impairment in awareness. She also had generalized tonic-clonic seizures, but this became less frequent with adjustment of her antiseizure drug regimen. She tried almost all antiseizure drugs in various combinations and at optimal dosages. She has also been on VNS therapy since the age of 29. Despite these measures, she continued to have breakthrough seizures almost every month. During her EMU evaluation, while off her antiseizure drugs, she had a focal frontal lobe seizure that evolved into a secondary generalized tonic-clonic seizure. Her interictal EEG showed focal spikes over the left and the right frontal head regions. The next day, her EEG showed sustained 2.5-3 Hz GSWDs (Figure 3). She appeared confused and her responses to questions were limited to one or two words. The NCSE lasted about 36 minutes and required a total of four doses of lorazepam 1 mg IV before finally stopping. Resumption of her antiseizure drugs prevented further seizures.

## Discussion

The presence of GSWDs on the EEG during an absence seizure is considered a hallmark of IGE. Ever since GSWDs were first reported [11], researchers have sought to understand the neural underpinnings of these distinctive EEG waveforms. According to the centrencephalic theory, GSWDs are triggered and synchronized by a pacemaker within the thalamus and brainstem reticular formation [12]. The corticoreticular theory argues that the hyperexcitable cerebral cortex generates GSWDs in response to otherwise normal thalamic excitation [13]. More specifically, GSWDs arise from the same thalamocortical circuits that normally produce sleep spindles [14]. The term “dyshormia” was later coined to describe GSWDs occurring during K-complexes in non-rapid eye movement (REM) sleep [15]. The cortical focus theory posits a focal cortical trigger for GSWDs and their subsequent generalization via synchronization of corticocortical and thalamocortical networks [16]. Simultaneous EEG and functional MRI (EEG-fMRI) studies showed that GSWD-related blood oxygenation level-dependent (BOLD) activations occurred earlier in the frontal cortex relative to the thalamus [17]. In addition, GSWD-related BOLD deactivations occurred in the default mode network (DMN) of the brain, providing a neural basis for the impairment of consciousness during absence seizure [18].

ASE is absence seizure with an arbitrarily defined duration of >10-15 minutes [6]. It is characterized by variable degrees of impairment in consciousness and by the appearance of 1-4 Hz GSWDs in the EEG. As a rule, ASE occurs in patients with IGE (exemplified by Patient 1), but ASE can also occur “de novo” in patients with no history of seizure or epilepsy (exemplified by Patient 2). Earlier cases of de novo ASE mainly involved older adults with altered mental status due to drug withdrawal, drug toxicity, or metabolic disturbances [19]. The mechanism of seizure perpetuation in ASE is poorly understood [8]. Some studies found a difference in cerebral blood flow and brain metabolism patterns between brief GSWDs versus very long GSWDs and ASE [20,21]. Spectral analysis of the EEG during late-onset ASE showed maximal spectral power in the anteromedial frontal cortex for the 12-14 Hz band (spike component of GSWDs) and in the frontal and temporal limbic cortex for the 1-6 Hz band (slow-wave component of GSWDs [22].

Although absence epilepsy has been viewed as the prototype for generalized epilepsy, particularly IGE, absence seizures may actually manifest some of the characteristics of focal seizures, particularly frontal lobe seizures [23]. A seizure focus was identified in rodents with genetic absence epilepsy, first in the primary somatosensory cortex [16], and later in the secondary somatosensory and adjacent insular cortex [24]. A seizure focus was also detected in the frontal neocortex of children with childhood absence epilepsy [25]. It is thought that the cortex is unevenly hyperexcitable in absence epilepsy with some regions responding to thalamic and brainstem inputs by generating spike-and-wave activity that are then propagated to other regions via commissural and thalamocortical pathways [26]. Not only is the trigger of absence seizure focal, but the selective recruitment of thalamocortical networks also restricts cortical activation to the mesiofrontal, orbitofrontal, and somatosensory cortices [27]. Although absence seizures are considered “non-motor”, there is ample evidence that motor signs are the rule, not the exception, in absence seizures [28,29].

The converse is also true: patients with focal epilepsy can manifest some of the characteristics of generalized epilepsy. For example, generalized epileptiform discharges can appear on the EEG of patients with frontal lobe epilepsy [30]. This phenomenon, known as secondary bilateral synchrony, implies that ictal activity is initiated in a frontal lobe focus and spreads rapidly, via callosal connections, to the contralateral frontal lobe [31,32]. Secondary bilateral synchrony rarely manifests as well-formed GSWDs. However, frontal lobe seizures with typical GSWDs that are indistinguishable from the GSWDs in IGE have been reported in the literature [33]. Some authors adapted the name “pseudo-absence” to describe frontal lobe seizures that mimicked absence seizures, including the configuration and brevity of the GSWDs [34,35]. The EEG-fMRI recording during “focal” frontal lobe seizures showed BOLD activation in the cingulate gyri on both sides, in addition to focal activations in the frontal cortex and thalamus [36]. Like absence seizures, BOLD deactivations in the DMN have also been observed during frontal lobe seizures and this may be the reason for the cognitive impairment in some frontal lobe seizures [9,36]. The intersection between frontal lobe epilepsy and IGE has been explored from the standpoint of dyshormia and the influence of sleep on ictogenesis [37,38].

Table 1 shows the application of the quadriaxial classification system to the status epilepticus of the three cases presented in this article [6].

**Table 1:** The quadriaxial system for classifying status epilepticus applied to the three cases presented in this article Source: Ref. [6]

	Patient 1	Patient 2	Patient 3
Axis 1 (semiology)	B. Nonconvulsive status epilepticus	B. Nonconvulsive status epilepticus	B. Nonconvulsive status epilepticus
	B.2. Without coma	B.2. With coma	B.2. Without coma
	B.2.a. Generalized	B.2.a. Generalized	B.2.b. Focal
	B.2.a.a. Typical absence status	B.2.a.a. Typical absence status	B.2.b.c. With impaired consciousness
Axis 2 (etiology)	Juvenile absence epilepsy	No history of seizure/epilepsy	Frontal lobe epilepsy
	Status epilepticus was precipitated by reduction in antiseizure drugs.	Status epilepticus occurred “de novo” in the setting of cerebral anoxia from cardiac arrest.	Status epilepticus was precipitated by reduction in antiseizure drugs.
Axis 3 (EEG correlate)	Generalized 2.5-3 Hz spike-and-wave discharges	Generalized 2.5-3 Hz spike-and-wave discharges	Generalized 2.5-3 Hz spike-and-wave discharges
Axis 4 (age of the patient)	Adult (44 years old)	Elderly (73 years old)	Adult (41 years old)

There is no issue with the axis 1 classification of status epilepticus in Patient 1. She had intractable juvenile absence epilepsy and developed ASE because her antiseizure drugs were reduced during her EMU evaluation. The situation with Patient 2 is different. She had no history of seizure or epilepsy, and NCSE occurred at a time when she was in coma because of anoxic brain injury. Her EEG showed 2.5-3 Hz GSWDs, a pattern consistent with “de novo ASE”, but “NCSE in coma” seems to be the proper status epilepticus diagnosis. NCSE is relatively common after anoxic brain injury, but NCSE with well-formed (i.e., typical) GSWDs appears to be rare after cerebral anoxia. We are aware of only two published reports of de novo ASE in the setting of an anoxic brain injury [39,40]. In both cases, as well as in Patient 2, the EEG showed well-formed GSWDs normally seen only in IGE. Is the axis 4 classification of “de novo absence status of later life” applicable to the case of Patient 2? Although initially reported in older adults, de novo ASE has been increasingly observed in children and non-elderly adults [41-44].

The case of Patient 3 presents yet another challenge in choosing a status epilepticus diagnosis. An axis 1 diagnosis for Patient 3 would be “focal frontal lobe NCSE without coma with impaired consciousness.” Although precise, such a long-winded diagnostic term is not pragmatic because it highlights the focal and ignores the generalized aspects of NCSE. Patient 3 is a case in point; she had focal epilepsy, but the onset of NCSE was unmistakably generalized, with GSWDs appearing abruptly in the EEG. A term that does not emphasize the focal or generalized aspects of NCSE may be more appropriate, for example, “dialeptic status epilepticus” [45]. This neutral term is unlikely to detract physicians from the fact that cognitive impairment in NCSE is often due to activation and/or deactivation of DMN structures in both hemispheres [18,36,37]. The focal-generalized dichotomization of seizures and epilepsy has confused physicians for decades. It has reinforced the notion of seizures as either focal or generalized even though most (if not all) seizures involve both focal and generalized elements. It is about time to address this issue by using pathophysiologically neutral terms like “dialeptic NCSE” and “absence NCSE” and avoiding terms like “complex partial status epilepticus” and “focal NCSE with impaired consciousness.”

## Conclusions

In this observational study, three different types of NCSE, ASE in a patient with IGE, NCSE in coma (with EEG features of de novo ASE) in a patient with no prior seizure or epilepsy, and NCSE with impaired consciousness in a patient with frontal lobe epilepsy, showed GSWDs in the EEG. This is yet another evidence of the fine line between absence and focal epilepsy. Absence seizures are considered generalized but have been shown many times to originate from a cortical focus. Frontal lobe seizures are focal by definition but may generalize rapidly without any trace of focality. Focal NCSE with impaired consciousness involves activation and/or deactivation of the default mode network in both hemispheres. It is time that we dismantle the stronghold of the focal-generalized dichotomy on the nosology of seizure and epilepsy.
